# Supplementary material for: Modified Protocol of Harvesting, Extraction, and Normalization Approaches for Gas Chromatography Mass Spectrometry-Based Metabolomics Analysis of Adherent Cells Grown Under High Fetal Calf Serum Conditions
Source: Metabolites. 2019 Dec 18;10(1):2. doi: 10.3390/metabo10010002 (PMC7023238; doi:10.3390/metabo10010002)
Supplement: Supplementary file 1 [file metabolites-10-00002-s001.pdf]

Supplementary material:

Modified protocol of harvesting, extraction and normalization approaches for gas chromatography mass spectrometry-based metabolomics analysis of adherent cells grown under high fetal calf serum conditions

Raphaela Fritsche-Guenther [1,2], Anna Bauer [1,2], Yoann Gloaguen [1,2,3], Mario Lorenz [4,5] and Jennifer A. Kirwan [1,2,*]

Table S1. Individual metabolite relative standard deviation (RSD) of HCT116 cells cultured in 10% and 20% FCS conditions using different quenching solvents, extraction ratios and polar volumes. *: phosphate.

**Condition name**	**50_LOW**		**50_MEDIUM**		**50_HIGH**		**80_HIGH**	
**FCS [%]**	**10**	**20**	**10**	**20**	**10**	**20**	**10**	**20**
**Biological replicates**	**4**	**5**	**3**	**3**	**4**	**4**	**5**	**4**
Alanine	83	38	13	35	32	27	28	55
Asparagine	69	111	8	48	27	48	19	43
Aspartic acid	95	32	2	39	20	29	14	49
Glutamine	69	49	32	51	6	25	23	16
Glycine	70	31	22	55	8	21	17	40
Isoleucine	63	36	35	70	24	24	20	16
Leucine	70	30	18	53	7	19	13	27
Lysine	72	131	48	87	50	29	55	58
Methionine	66	66	48	93	41	27	48	15
Phenylalanine	67	30	20	58	6	23	13	25
Proline	67	29	21	70	7	21	31	57
Serine	68	47	24	67	20	20	21	21
Threonine	105	37	9	30	16	29	29	63
Tryptophan	42	70	34	63	52	22	32	38
Tyrosine	68	62	19	54	18	66	42	61
Valine	67	31	20	62	8	19	13	63
Fructose-6-phosphate		39	40	70		67	25	86
Glucose-6-phosphate	73	56	24	61	21	35	30	59
Glyceric acid-3-P*	90	32	16	59	34	74	112	23
Phosphoenolpyruvic acid	117	27	10	50	11	31	129	43
Pyruvic acid	68	58	27	63	25	57	30	25
Dihydroxyacetone P*		38		95				
Glycerol	66	32	18	49	4	18	15	12
Glycerol-3-phosphate	64	29	32	54	10	20	25	24
Adenine	73	32	21	54	8	26	28	12
Adenosine	155	103	131	63	35	77	103	15
Uracil	65	36	29	66	16	26	22	26

Butanoic acid, 3-hydroxy-	73	35	26	63	40	23	18	3
Butanoic acid, 4-amino-	63	31	16	57	7	27	20	51
Erythritol	66	32	20	54	9	21	12	24
Glutaric acid	64	29	34	54	12	19	11	29
Glyceric acid	152	31	23	133	11	50	12	50
Ribose	75	44	26	33	12	22	25	22
Ribose-5-phosphate		33		37		78		47
Citric acid	67	31	20	49	105	21	83	181
Fumaric acid	63	32	24	57	12	27	22	28
Glutaric acid, 2-hydroxy-	91	30	25	48	25	48	54	44
Glutaric acid, 2-oxo	66	34	28	50	10	26	23	28
Malic acid	68	32	22	55	8	20	14	30
Succinic acid	66	32	21	54	9	21	14	29
Glutamic acid	72	33	15	66	15	27	46	98
**Median RSD**	**68**	**33**	**22**	**55**	**14**	**26**	**23**	**30**

Table S2. Individual metabolite relative standard deviation (RSD) of HUVECs cultured in 20% FCS conditions using different quenching solvents, extraction ratios and polar volumes for the different protocols. Cells were measured in different batches (dashed line). *: 50_Medium.

**Condition name**	**50_LOW**	**50_MED***	**50_MED***	**80_HIGH**	**50_HIGH**	**80_HIGH**
**Biological replicates**	**2**	**3**	**3**	**3**	**2**	**3**
Alanine	41	22	39	16	27	26
Asparagine					8	84
Aspartic acid	51	75	41	33	39	55
Glutamine					19	85
Glycine	13	18	42	21	4	24
Isoleucine		40	44	59	55	17
Leucine		49	43	59	7	79
Lysine	51	50	34	10	9	111
Methionine	18		31	51	42	73
Phenylalanine	9	21	35	28	7	56
Proline	121	73	47	13	32	
Serine	50	34	41	39	8	61
Threonine	8	67	33	31	14	14
Tyrosine						147
Valine	17		44	17	6	71
Glyceric acid-3-phosphate					23	54
Lactic acid	10	11	31	17	7	8
Phosphoenolpyruvic acid	98		77	16	6	37
Pyruvic acid	9	2	48	32	6	9
Glycerol	4	11	36	13	4	5
Glycerol-3-phosphate	26	50	45	13	35	46
Adenine	11	27		44	1	35
Adenosine		26	35	17		
Cytosine	29		50	5		

Uracil			31	19		120
Butanoic acid, 3-hydroxy-	19	13	38	8	20	16
Butanoic acid, 4-amino-					49	137
Erythritol	10	15	39	12	12	9
Glutaric acid	26		15	12	21	15
Glyceric acid	22	16	31	8	43	36
Ribose	13	22	39	34	10	21
Ribose-5-phosphate			29			69
Citric acid	36	63	38	15	11	36
Fumaric acid		51	38	4	85	7
Glutamic acid	29		96	59	21	74
Glutaric acid, 2-hydroxy-	1	37	67	18	19	26
Glutaric acid, 2-oxo-	7	5		33	5	15
Malic acid	32	25	39	5	1	14
Succinic acid	16	15	32	11	31	10
**Median RSD**	**18**	**26**	**39**	**17**	**13**	**36**

Table S3. Median of the relative standard deviation (RSD) per metabolite of HUVECs cultured in 20% FCS conditions using different quenching solvents, extraction ratios and polar volumes for the different protocols. Cells were measured in different batches.

**Condition name**	**50_LOW**	**50_MEDIUM**	**50_HIGH**	**80_HIGH**
**Biological replicates**	**2**	**3**	**2**	**3**
**Male HUVEC**	18%	26%		
**Female HUVEC**		39%		17%
**Female HUVEC**			13%	36%

Table S4. Percentage of metabolites with a relative standard deviation (RSD) < 30% of HUVECs cultured in 20% FCS conditions using different quenching solvents, extraction ratios and polar volumes for the different protocols. Cells were measured in different batches.

**Condition name**	**50_LOW**	**50_MEDIUM**	**50_HIGH**	**80_HIGH**
**Biological replicates**	**2**	**3**	**2**	**3**
**Male HUVEC**	68%	54%		
**Female HUVEC**		6%		67%
**Female HUVEC**			74%	44%

Table S5. Relative standard deviation (RSD) of measured protein amount of HUVECs cultured in 20% FCS conditions using different quenching solvents, extraction ratios and polar volumes for the different protocols. Cells were measured in different batches.

**Condition name**	**50_LOW**	**50_MEDIUM**	**50_HIGH**	**80_HIGH**
**Biological replicates**	**2**	**3**	**2**	**3**
**Male HUVEC**	49%	15%		
**Female HUVEC**		38%		9%
**Female HUVEC**			19%	9%

Table S6. Median of the relative standard deviation (RSD) per metabolite and percentage of metabolites with a RSD < 30% of HUVECs (three biological replicates) cultured in 20% FCS conditions.

**Normalization Strategy**	**Cell mass**	**Protein**
**Median RSD [%]**	61%	52%
**RSD < 30% [%]**	18%	26%

Table S7. List of metabolite derivatives and their biological group used for reference search. AA: Amino acids. PPP: Pentose phosphate pathway. TCA: Tricarboxylic acid cycle. TMS: Trimethylsilyl derivatives. MeOX: Methoxyamine hydrochloride.

Group	Metabolite	Detected as
AA	Alanine	3TMS
		2TMS
AA	Asparagine	2 TMS
AA	Aspartic acid	2 TMS
		3 TMS
AA	Cysteine	3 TMS
AA	Glutamine	3TMS
AA	Glycine	2TMS
		3TMS
AA	Isoleucine	1TMS
		2TMS
AA	Leucine	1TMS
		2TMS
AA	Lysine	3TMS
AA	Methionine	1TMS
		2TMS
AA	Phenylalanine	1TMS
		2TMS
AA	Proline	1TMS
		2TMS
AA	Serine	2TMS
		3TMS
		4TMS
AA	Threonine	2TMS
		3TMS
AA	Tryptophan	2TMS
AA	Tyrosine	3TMS
AA	Valine	1TMS
		2TMS
Glycerol	Dihydroxyacetone phosphate	1MeOX 3TMS
Glycerol	Glycerol	3TMS
Glycerol	Glycerol-3-phosphate	4TMS
Glycolysis	Fructose-6-phosphate	1MeOX 6TMS
Glycolysis	Glucose-6-phosphate	1MeOX 6TMS
Glycolysis	Glyceric acid-3-phosphate	4TMS

Glycolysis	Lactic acid	2TMS
Glycolysis	Phosphoenolpyruvic acid	3TMS
Glycolysis	Pyruvic acid	1MeOX 1TMS
Nucleobase	Adenine	2TMS
Nucleobase	Uracil	2TMS
Nucleosid	Adenosine	3TMS
Nucleosid	Adenosine	4TMS
Nucleosid	Cytosine	2TMS
Others	Butanoic acid, 3-hydroxy-	2TMS
Others	Butanoic acid, 4-amino-	3TMS
Others	Erythritol	4TMS
Others	Glutaric acid	2TMS
Others	Glyceric acid	3TMS
PPP	Ribose-5-phosphate	1MeOX 5TMS
PPP	Ribose	1MeOX 4TMS
TCA	Citric acid	4TMS
TCA	Fumaric acid	2TMS
TCA	Glutamic acid	2TMS
		3TMS
TCA	Glutaric acid, 2-hydroxy-	3TMS
TCA	Glutaric acid, 2-oxo-	1MeOX 2TMS
TCA	Malic acid	3TMS
TCA	Succinic acid	2TMS

Table S8. Technical variation during gas chromatography mass spectrometry (GC-MS) run of four pooled samples. Alkane 32 (C32H66, Dotriacontane) is an acyclic saturated hydrocarbon containing 32 carbons. It is used to determine the instrument stability over time. Alkane 32 is measured independently of any extraction or derivatization steps. RSD: Relative standard deviation.

	**RSD**
**Sum of area**	10%
**Alkane 32**	15%
**Internal standard**	18%

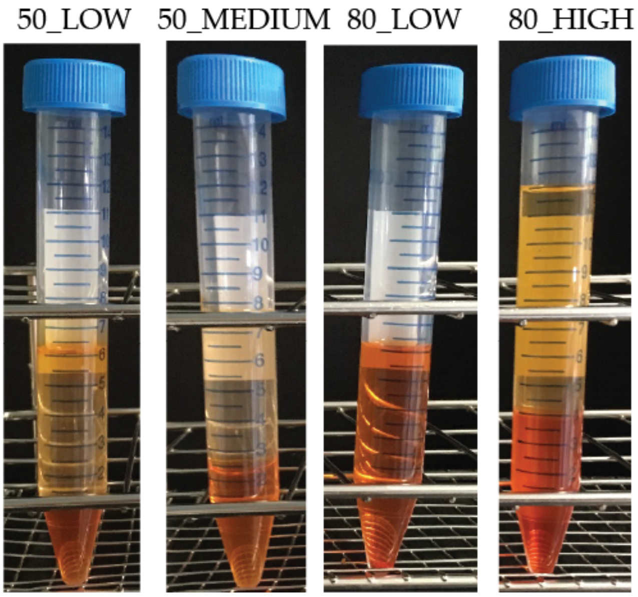

Figure S1. 50% and 80% of MeOH quenching buffer and different ratios of MeOH:$CHCl_3$:H_2O were mixed for extraction steps. Sudan I was added to aid visualization of phase separation. No phase separation could be observed using 80_LOW condition (1.0 mL $CHCl_3$ for extraction resulting in a final volume of 6.0 mL). Therefore, the condition was not used for further testing.

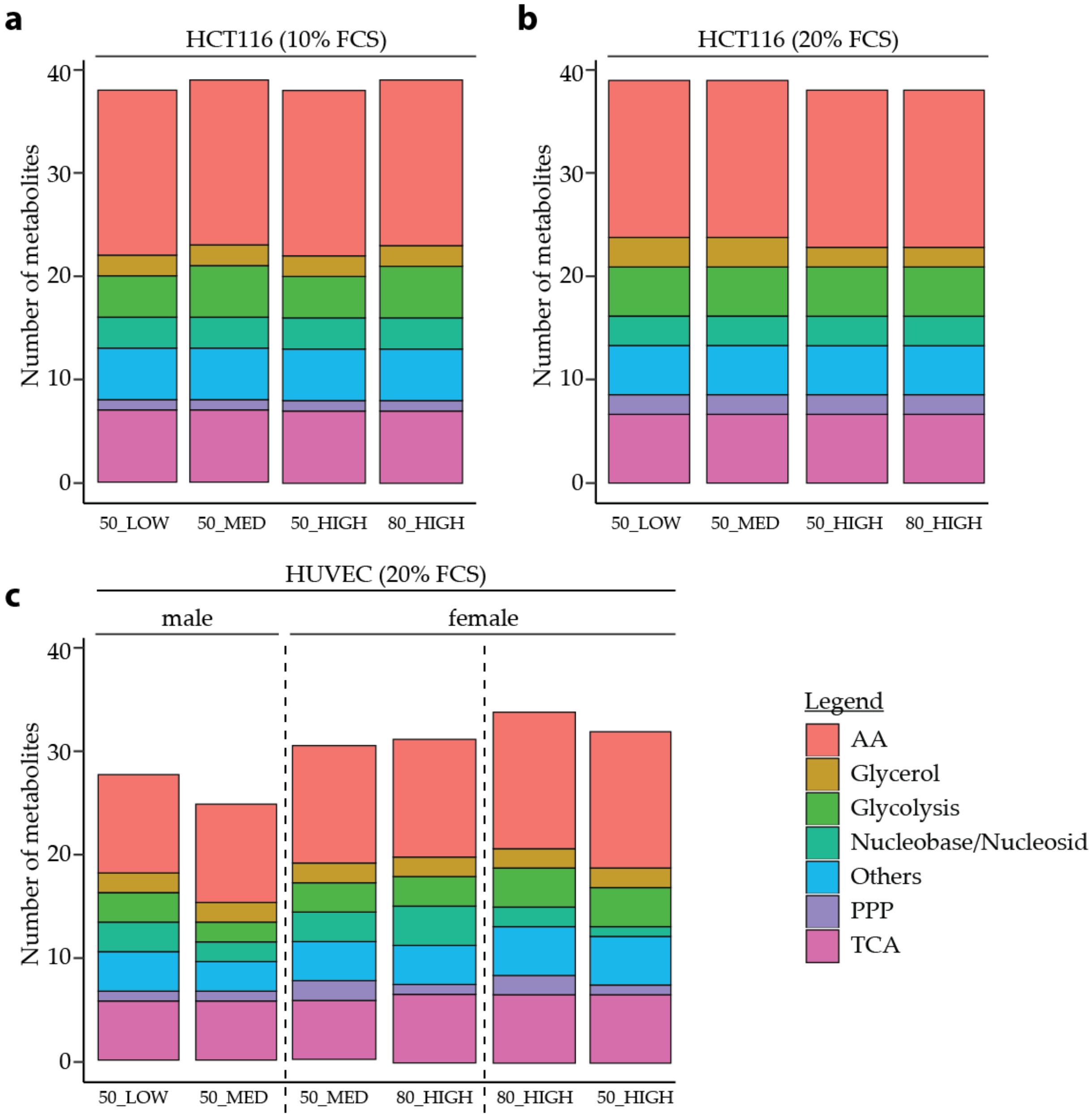

Figure S2. Recovery of annotated metabolites per biological group for different quenching and extraction methods. **(a)** HCT116 cells were cultivated in 10% FCS (in minimum three out of five biological replicates). **(b)** HCT116 cells were cultivated in 20% FCS (in minimum four out of five biological replicates). **(c)** HUVECs were cultured in 20% FCS (in minimum two out of three biological replicates). HCT116 cells were measured in one batch while HUVECs were measured in different batches (dashed line). The column data represents the number of annotated metabolites for each protocol. AA: Amino acids. PPP: Pentose phosphate pathway. TCA: Tricarboxylic acid cycle.

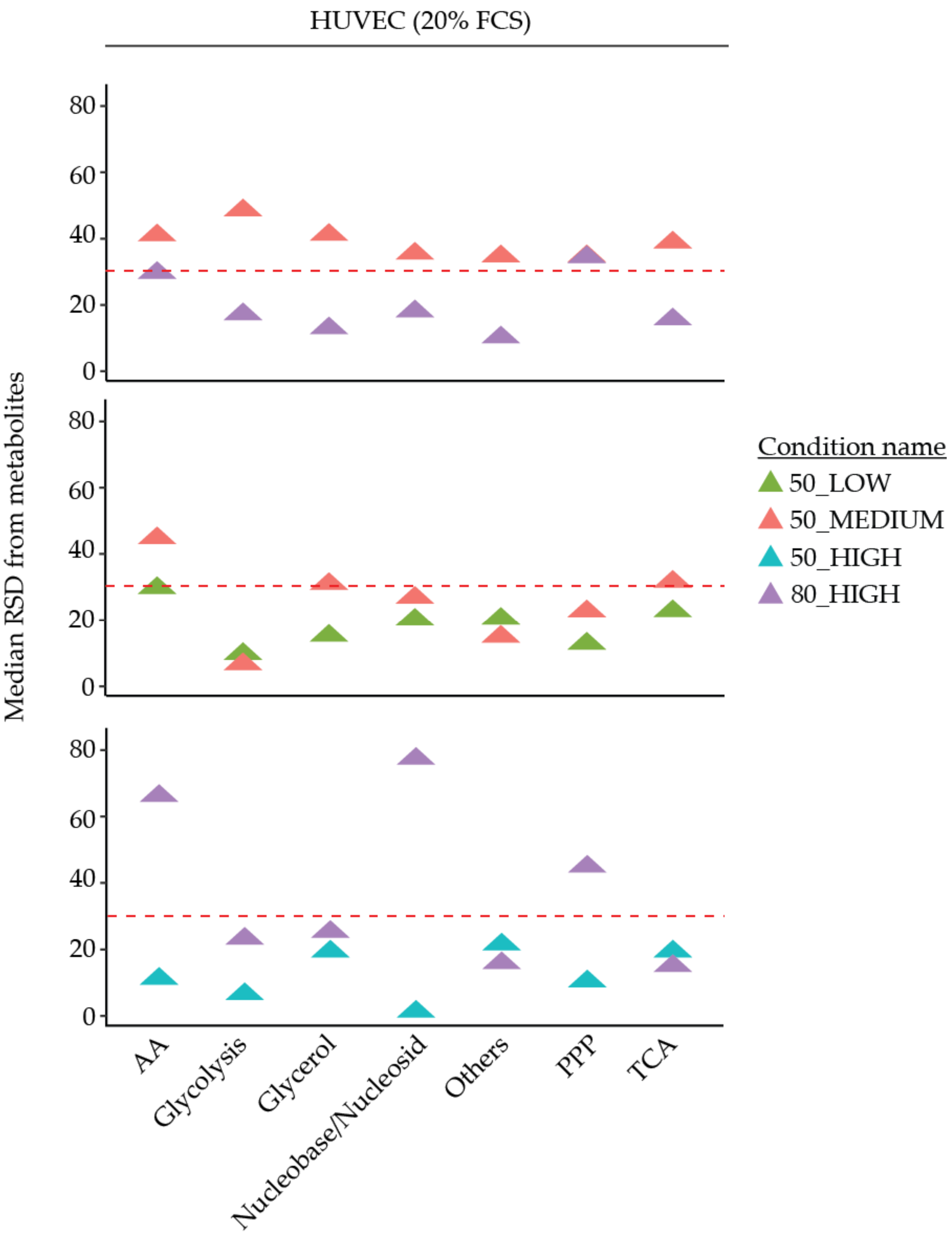

Figure S3. Median relative standard deviation (RSD) of individual metabolites separated by metabolite classes from in minimum two out of three replicates of HUVECs cultured in 20% FCS conditions using different quenching solvents, extraction ratios and polar volumes. The dashed line represents the maximum 30% RSD threshold advised by the Food and Drug Administration (FDA). Cells were measured in different batches reflected by the three graphs. AA: Amino acids. PPP: Pentose phosphate pathway. TCA: Tricarboxylic acid cycle.

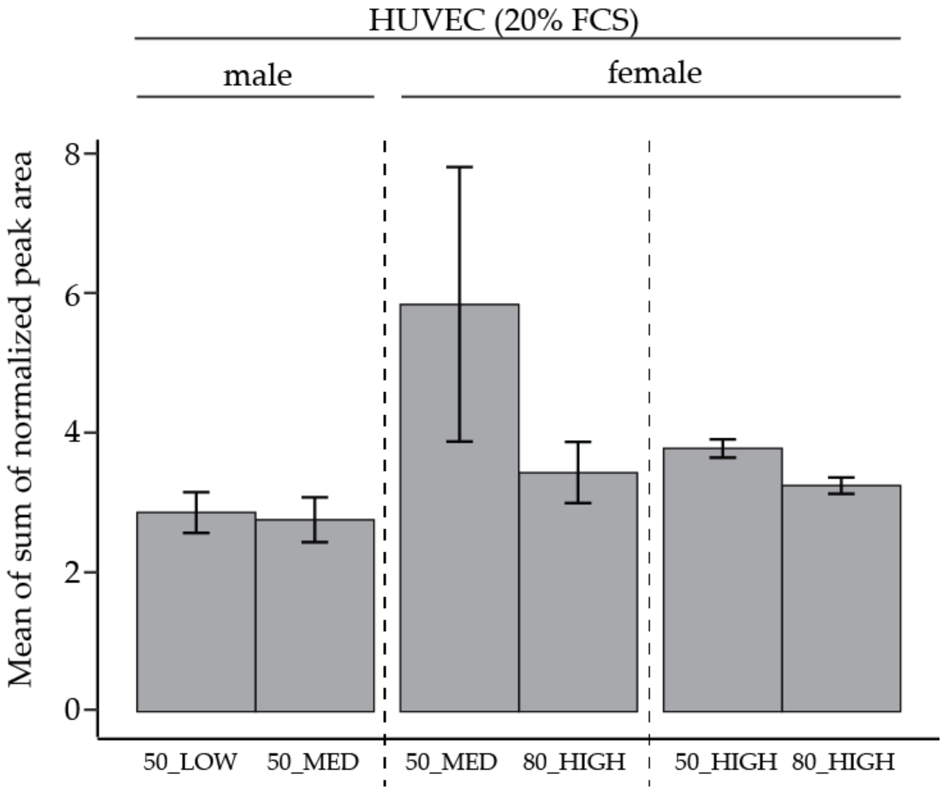

Figure S4. Comparison of the mean of sum of normalized peak area for different quenching and extraction methods. HUVECs were cultured in 20% FCS. The cells were measured in 2 different batches (dashed line). Data from in minimum two out of three biological replicates. The peak area was normalized to cinnamic acid and protein amount. Due to the low number of biological replicates no significances were measured.

Berlin Institute of Health (BIH) Metabolomics Platform, Berlin, Germany

File S1. Standard operating protocol for harvest and extraction of adherent cells grown under 10% or 20% fetal calf serum (FCS) conditions version 1.

If you use this protocol please cite as: Fritsche-Guenther et al., "Modified protocol of harvesting, extraction and normalization approaches for gas chromatography mass spectrometry-based metabolomics analysis of adherent cells grown under high fetal calf serum conditions", Metabolites 2019

This protocol has been validated with respect to quenching buffers and extraction solvents using HUVEC and HCT116 cells. Other variables e.g. wash buffers, cell culture conditions, media changes etc. will be cell-type dependent and need to be determined by the user.

Researchers should familiarize themselves with the health and safety implications of the procedures and chemicals they will be using and take appropriate precautions as necessary in consultation with the Health and Safety department of their institution. Solvents should be GC-MS grade or higher. Prepare all necessary equipment and solvents in advance. Use the same buffers, solvents and batch of vials for any individual study.

To reduce the technical variability of this method, it is important that cells are harvested quickly and reproducibly using ice cold methanol (MeOH). To ensure these conditions, it is recommended that no more than three culture plates are harvested at a time.

Sample type

Adherent cell lines or primary cells plated in 10 cm^2 dishes. Confluency at day of harvest should be 80-90%.

Equipment (prepare in advance)

- Appropriate working area in accordance with your local health and safety guidelines e.g. fume hood or solvent safe microbiologic cabinet
- 15 mL Falcon (2 per cell culture plate)
- 5 mL pipette and tips (do not use a pipette controller or a pump for media removal)
- Crushed ice
- Cell scraper (uncover cell scraper prior to use and store in completely clean glass beaker)
- Centrifuge (cooled to 4°C)
- Rotational vacuum concentrator

Solvents

- **Washing buffer** (140 mM sodium chloride, 5 mM 4-(2-hydroxyethyl)-1-piperazineethanesulfonic acid = HEPES, pH 7.4) + supplements (depend on the growth media conditions) **pre-warmed to 37 °C**. Suggested maximum storage time for washing buffer is 2 months, however, check before experiment if the buffer is cloudy; if so, prepare new.
- **50% MeOH** in water (contains 2 µg/mL cinnamic acid) **pre-chilled to -20 °C**. When in use, the solution should be stored on ice to keep it cold. Maximum time the MeOH solution can be used should be 30 min to avoid it warming.
- 100% MeOH
- Chloroform ($CHCl_3$)
- Water (H_2O)

Protocol

<u>*Harvest*</u>

1. Wash cells rapidly (20 seconds) with 5 mL of washing buffer.
2. Quench cells by using 5 mL of ice-cold 50% MeOH.
3. Immediately scrape cells into the MeOH solution and collect the methanolic lysates into a 15 mL Falcon. Samples can be stored at -80 °C until extraction.

<u>*Extraction*</u>

4. For extraction add 4 mL of $CHCl_3$, 1.5 mL of 100% MeOH and 1.5 mL of H_2O to the methanolic cell extracts.
5. Shake extracts for 60 min at 4 °C.
6. Centrifuge extracts at 4,149 x g for 10 min at 4 °C to separate the phases.
7. Collect 6 mL of the upper polar phase and transfer to a new 15 mL Falcon.
8. Create a pooled quality control (QC) sample by pooling the leftover polar phase from each sample. The amount of pooled QC required is dependent on the number of samples to be analysed and your QC regime (recommended two at the beginning of the run, two at the end and every 5 to 10 samples). The pooled QC should then be divided into 6 mL aliquots for further extraction. 6 mL is enough to create two QC samples. This pooled sample is then extracted alongside the other samples and used as quality control for technical variability of the extraction process and instrument.

   OPTIONAL: if you wish to measure total protein content, keep the original Falcon tube and follow steps 10 to 14.

9. Dry the polar phase at 30 °C at a speed of 1,550 x g at 0.1 mbar using a rotational vacuum concentrator. Samples can be stored at -80 °C until backup generation or GC-MS measurement.

   OPTIONAL: To generate backup samples (and to generate two QCs from a single extraction), samples can be split before being dried down, or can be resuspended and split after drying down. The latter method can have some advantages if you are limited in space in your rotational vacuum concentrator.

<u>*Measurement of total protein*</u>

10. The original Falcon tube should now have a protein pellet and a $CHCl_3$ mix left. The total protein amount can be measured from this if required for normalization. If this is required, add 8 mL of 100% MeOH to what remains in the Falcon tube.
11. Vortex for 10 sec and centrifuge at 16,000 x g for 10 min.
12. Carefully discard the supernatant.
13. Air dry the pellet at room temperature for 30 min.
14. Resuspend the pellet in an appropriate buffer and determine the protein amount using your favored method. We resupend the pellet in 8 M urea buffer (in 50 mM HEPES, pH 8.5) and determined the protein concentration using a bicinchoninic acid (BCA) assay (Thermo Fischer Scientific, Waltham, Massachusetts, US) following the manufacturer`s instructions.
